# Multiparameter Space Decision Voting and Fusion Features for Facial Expression Recognition

**DOI:** 10.1155/2020/8886872

**Published:** 2020-12-29

**Authors:** Yan Wang, Ming Li, Xing Wan, Congxuan Zhang, Yue Wang

**Affiliations:** ^1^College of Automation Engineering, Nanjing University of Aeronautics and Astronautics, Nanjing 210016, China; ^2^Key Laboratory of Jiangxi Province for Image Processing and Pattern Recognition, Nanchang Hangkong University, Nanchang 330063, China; ^3^China Union Network Communication Co., Ltd., Jiangxi Branch, Nanchang 330029, China

## Abstract

Obtaining a valid facial expression recognition (FER) method is still a research hotspot in the artificial intelligence field. In this paper, we propose a multiparameter fusion feature space and decision voting-based classification for facial expression recognition. First, the parameter of the fusion feature space is determined according to the cross-validation recognition accuracy of the Multiscale Block Local Binary Pattern Uniform Histogram (MB-LBPUH) descriptor filtering over the training samples. According to the parameters, we build various fusion feature spaces by employing multiclass linear discriminant analysis (LDA). In these spaces, fusion features composed of MB-LBPUH and Histogram of Oriented Gradient (HOG) features are used to represent different facial expressions. Finally, to resolve the inconvenient classifiable pattern problem caused by similar expression classes, a nearest neighbor-based decision voting strategy is designed to predict the classification results. In experiments with the JAFFE, CK+, and TFEID datasets, the proposed model clearly outperformed existing algorithms.

## 1. Introduction

Facial expressions, as a form of nonverbal communication, convey social information among humans and are regarded as an emotional measurement that can be used to understand human actions and behaviors [1]. In the computer vision field, the recognition of static-based and dynamic-based facial expressions is widely used in various applications, such as e-learning [2], driver drowsiness estimation [3], and pain assessment [4].

Generally, facial expression recognition (FER) has four crucial steps: face detection, face image preprocessing, facial feature extraction, and classification [5]. The facial expression representation depends on facial muscle movements. For example, static facial expression images intuitively exhibit deformations of facial components and skin appearance texture changes of expressions. Holistic feature extraction methods are mainly categorized as geometric-based and subspace learning-based feature extraction. The well-known Facial Action Coding System (FACS) was first proposed by Ekman and Friesen [6]. FACS is a facial expression coding system that postulates six primary emotions that are composed of a set of facial muscle action units (AUs). In addition, each expression is represented by a particular combination of specific AUs. However, the unit modules are complex and the facial expression features are selected by manual intervention to some extent. Hence, automatic feature point location and feature extraction methods have followed. The Active Shape Model (ASM) with local texture information [7] and the Active Appearance Model (AAM) [8] with global texture information are both geometric-based models. In general, the initialization of facial landmarks depends on manual annotation, which may lead to expensive labor costs. The state-of-the-art AAM and its derived algorithms for FER focus on how to locate the fiducial points at the appropriate facial positions [9], which need to accurately extract parameter features, i.e., appearance parameters, shape parameters, and texture parameters [10].

Many research works focus on combination methods to achieve a high recognition rate. Holistic feature subspace methods mainly include principal component analysis (PCA) [11], Fisher linear discriminant analysis (FLDA) [12], manifold learning [13], and graph embedding [14], which are commonly combined with texture feature extraction methods, i.e., the Gabor wavelet [15], LBP [16], Haar-like [17], and HOG [18] features, for FER. Piparsaniyan et al. [19] used PCA to reduce the dimension of Gabor features. Han and Ming [20] employed a combined strategy of LBP feature extraction and Supervised Locality Preserving Projection (SLPP) dimension reduction for facial expression recognition. In addition, Sun and Yu [21] combined Gabor features and LBP features to represent facial expression features.

To elaborately extract facial expression features, some studies have divided facial images into nonoverlapping blocks [20–22]. Because expression features reflect the changes of the direction, edge, and intensity of the texture of an image, extracting features in regions of interest (ROIs) is a common practice [23–25]. Most ROIs are eye, mouth, and eyebrow regions, which are fixed in a set of sizes. However, the features extracted from these ROIs are inaccurate in general due to image misalignment. Moreover, the expression representations are variable according to the affections of different cultures; therefore, these features in fixed sizes of ROIs cannot well represent the intensity of expression changes. Furthermore, local texture feature extraction from these ROIs would lose some important information of the expressions.

As a global structural face descriptor, the MB-LBP descriptor was first proposed to describe face changes for face detection [26]. Hence, MB-LBP feature extraction is widely used in many face recognition applications [27–29]. Martínez-Díaz et al. [29] proposed MB-LBP features-based dissimilarity representations for face detection in which MB-LBP was employed to compare the average grayscale of the central rectangle with the average grayscale of a 3 × 3 neighborhood. Girish et al. [30] extracted MB-LBP features with different operator sizes from several blocks divided by user-defined sizes, and then, the combined MB-LBP histograms of blocks were used as features. Thus, MB-LBP is often utilized as a description of a face image to extract feature information and analyze the effect of the size of a neighborhood window [31].

Although the MB-LBP model with some special parameters can describe face changes, it may lose the details of local texture changes. An HOG descriptor [32] can be employed to extract the local texture features of facial components. It has been demonstrated that the HOG descriptor has a unique superior performance at representing appearances and shapes of expressions compared to other descriptors. To extract both the structural and local texture features of facial expressions to achieve high accuracy and stable robustness of FER, we propose a novel multiparameter feature space model in which MB-LBPUH and HOG features are fused together to represent facial expression features. Specifically, we design a nearest neighbor-based decision voting strategy for prediction. The new voting strategy increases the recognition accuracy and resolves the inconvenient classifiable pattern problem caused by similar expression classes.

In this paper, the proposed model is composed of multiparameter feature spaces. In each space, the various facial expressions are represented by fused MB-LBPUH and HOG features. From a decision perspective, our model provides a nearest neighbor-based decision voting strategy. The major contributions of this paper are as follows:

1. MB-LBPUH scale parameters of different expressions are selected based on the cross-validation recognition accuracies, which enhance the global feature discrimination of the expression structure.
2. Multiparameter facial expression feature spaces, in which a query sample can find its best match in different spaces, are built using LDA according to the various selected MB-LBPUH parameters.
3. A nearest neighbor-based decision voting strategy is designed to predict the classification results. The integrated predictive model can not only increase the recognition accuracy but also resolve the inconvenient classifiable pattern problem caused by similar expression classes.

The remainder of this paper is organized as follows.  Section 2 introduces related work on feature extraction and identification of FER.  Section 3 introduces the multiparameter feature space model and decision voting strategy.  Section 4 analyzes and discusses the experimental results.  Section 5 concludes the paper.

## 2. Related Work

FER has received wide attention because of the importance of human emotion in artificial intelligence. Several studies have been conducted on the topic of facial expression recognition. According to state-of-the-art FER research, they are classified as global feature extraction, local appearance feature extraction, fusion feature extraction, and classification.

- *Global Feature Extraction*. Global feature extraction has two major categories, namely, geometric feature extraction and geometric combined with appearance feature extraction. These types of methods describe face deformations intuitively and have low computational complexity. However, in these methods, it is difficult to mark the points of facial expression shapes. To address this issue, Sadeghi et al. [33] used a fixed geometric model to normalize facial images and extracted LBP features from mouth and eye patches to obtain local texture features. Cheon and Kim [34] extracted different AAM features between an input face image and a neutral expression face image to effectively represent the variations of expressions. Ren and Huang [35] utilized multipose AAM templates to estimate the poses and locate the feature points of facial expression images. To further describe the feature points, they [35] combined AAM with the SIFT descriptor to represent a hybrid facial expression feature.
- *Local Appearance Feature Extraction*. Local appearance feature extraction mainly includes histogram-based feature extraction and wavelet-based feature extraction. The well-known LBP and its variants are demonstrated to be efficient texture feature descriptors [16]. In addition, local directional patterns (LDP) [36] and local transitional patterns (LTP) [37] are also widely employed in FER. Polytypic multiblock local binary patterns (P-MLBPs) are proposed in [38] for automatic 3D FER. Wang et al. [39] adopted the wavelet coefficients of the discrete wavelet as facial expression features. Zhang et al. [40] utilized biorthogonal wavelet entropy to extract multiscale features and employed a stratified cross-validation model to obtain a good classification performance. However, histogram-based methods lose the structural information and the relationships of pixels and only reflect the statistical information of features. In addition, while wavelet-based methods can process images without any information loss, the computational complexity is high.
- *Fusion Feature Extraction*. A fusion feature includes more useful information than a single feature. The fused features are complementary, and the new fusion feature has better discrimination. Tariq and Huang [41] implemented different combination strategies to demonstrate the performance of various features, such as LBP, DCT, LPQ, and SIFT, combined with classifiers. The best classification result was obtained by all the classifiers with the four features fused together. To solve the lack of expression shape and contour information, Wang et al. [42] fused the weber local descriptor (WLD) and HOG features to form a new feature representation. To obtain a hybrid feature, Luo et al. [43] proposed a new feature combination of a PCA feature with an LBP feature, which included not only global image information but also local feature information. Sun et al. [44] proposed a hierarchical classification framework in which a fusion strategy that consisted of feature-level and decision-level fusion was applied to extract multimodal features.
- In addition, the emergence of a model-based fusion method, such as Canonical Correlation Analysis (CCA), improves the correlation between two features. Turan and Lam [45] employed the CCA algorithm to fuse the LPQ and PHOG features extracted from eyes and mouth window regions to maximize the correlation of the two features. El-Shazly et al. [46] employed three common transformations (FFT, DCT, and DWT) as feature descriptors and fused them using CCA. The experimental results showed that the performance of the fusion features is better than that of one kind of transform domain feature.
- *Classification*. To identify the category of facial expressions, various classification approaches have appeared, such as the Nearest Neighbors (NN) [47], *k*-Nearest Neighbors (KNN) [48], Sparse Representation-based Classification (SRC) [49], Support Vector Machine (SVM), and random forest and decision tree [50]. Decision level fusion integrates all kinds of measurement information to achieve a more accurate classification accuracy [51]. Yeom [52] stated that decision-level fusion is a high-level data fusion technique that includes max, averaging, and majority voting fusion rules. In addition, some decision-level fusion methods, such as Bayesian estimation and D-S evidential reasoning, are often implemented for classification [53].

Although the recognition rate of FER has led to great achievements, the discussion between hand-crafted features and deep features is still ongoing. The convolutional neural network (CNN) is widely used in FER tasks due to its automatic understanding. A significant peculiarity of the CNN is that it pays more attention to the local features of the target with a deep network. Yang et al. [54] proposed a double-channel CNN model to extract expression-related local features from LBP facial images and grayscale images. Xie and Hu [55] designed two individual CNN branches. One branch extracts holistic features from a whole image, and the other branch extracts local features from overlapped image patches.

To learn more particular features for facial expression representation, a deep architecture named the AU-inspired Deep Network (AUDN) [56], which is based on multiple facial action units (AUs), is designed to learn better features specific to expression representation. Kim et al. [57] proposed a hierarchical deep neural network in which two features are fused together to form a new feature representation. The new feature specifically interpreted as one feature is first extracted from the appearance feature-based network and then combined with geometric features in the hierarchical structure. In addition, to achieve a high recognition accuracy, feature learning [58] and different joint representations of features [59] are also used in their own network model. However, deep learning needs a number of samples for training to avoid overfitting. Although some deep learning approaches for FER fuse some low-level features to represent expressions, these features are distributed in various network channels, which increases the complexity of the model. It is particularly difficult to determine what the prediction relies on and which features play important roles. Furthermore, a network model has many parameters that need to be fine-tuned to achieve a satisfactory recognition accuracy. Our proposed model has a simple architecture with a low computation complexity, and its features are explainable.

## 3. Methods

### 3.1. General Framework

**Figure 1:**
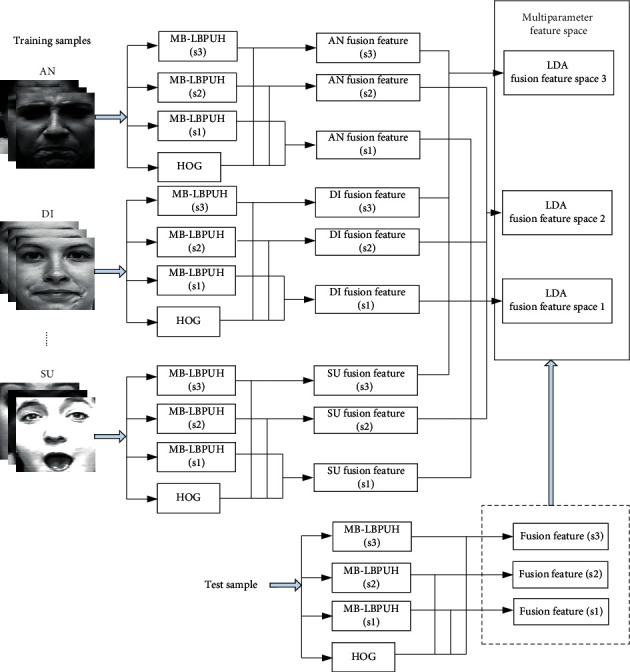
Framework of the proposed method.

Feature level fusion is a feature recombination according to the properties of the extracted features for improving recognition accuracy. The framework of the proposed model is shown in Figure 1. First, an MB-LBPUH feature and an HOG feature are extracted from training samples. In the MB-LBPUH feature extraction, the MB-LBPUH parameters are selected to build the feature space. Then, in the parameter feature spaces, a new representation of a facial expression is composed of MB-LBPUH and HOG features, namely, a fusion feature. Accordingly, we use LDA to reduce the dimension of the fusion feature. Finally, NN-based decision voting is applied to these feature spaces for prediction.

In the next two sections, we provide details of MB-LBPUH and the HOG feature extraction algorithm, respectively.

### 3.2. Facial Expression Feature Extraction

#### 3.2.1. MB-LBPUH Feature Extraction

Regardless of whether LBP or its improved operator is used, the main problem is that their operating space support is very small, which makes the binary mode between two pixels more vulnerable to the interference of subregion noise. In addition, the traditional LBP compares the eight neighboring pixels around a center pixel and encodes the binary values according to the comparison result. The 3 × 3 neighbor pixels are fixed; therefore, they do not capture the large-scale structural features of facial expressions. However, MB-LBP overcomes the shortcoming of the traditional LBP. MB-LBP utilizes the average of changeable subregion blocks to replace pixels, which has several advantages: (1) improving the robustness to noise, (2) encoding the image macrostructure and reflecting the image texture microstructure, and (3) operating the whole image and retaining the holistic feature.

**Figure 2:**
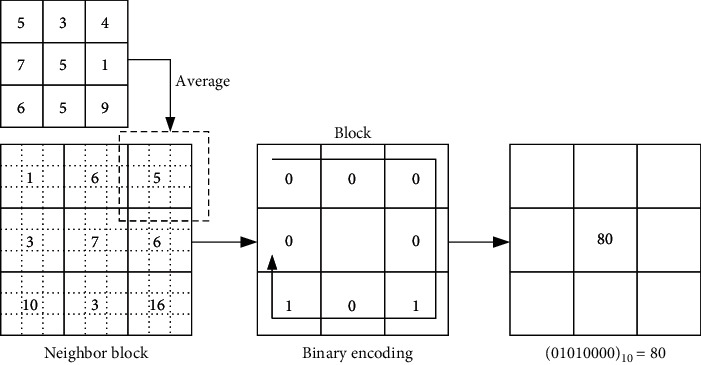
MB-LBP feature encoding.

In the original LBP, the common operator compares the center pixel with its 3 × 3 neighborhood pixels. However, the MB-LBP algorithm compares the average grayscale value of a center block with the average grayscale values of its neighborhood blocks (illustrated in Figure 2). The whole compared regions consist of eight neighboring blocks and one center block. Each subregion is a square block including (2*n*+1) × (2*n*+1) pixels, where *n* is an integer. Through comparison, a set of binary values are encoded by MB-LBP. Furthermore, if the average value of one neighborhood block is less than the average of the center block, then the binary value of the compared neighborhood block is encoded as 0; otherwise, the binary value is set as 1. Consequently, the method has a string of binary values of compared blocks in the clockwise direction. A decimal value corresponding to the string of binary values that represents the given pixels is then calculated.

In addition, the scale parameter *s*=2*n*+1 of the MB-LBP descriptor is important for describing texture changes, especially deformable textures. Once an appropriate scale parameter is determined, the extracted MB-LBP features can reflect some unique properties. When using the MB-LBP descriptor to filter a facial image, the MB-LBP features reflect not only the macrostructure of a facial texture but also the microstructure of the deformable texture of the expression. Therefore, the MB-LBP descriptor provides a more complete structural facial expression representation than the original LBP descriptor.

Briefly, the MB-LBP is defined as follows:

(1) $$\begin{array}{c} \text{MB}\_{\text{LBP}}_{{\bar{g}}_{\text{block}-c}}=\sum_{p=0}^{7}t\left({\bar{g}}_{\text{block}-p}-{\bar{g}}_{\text{block}-c}\right), \end{array}$$

where ${\bar{g}}_{\text{block}-p}=1/s^{2}\sum_{i=1}^{s^{2}}g_{\text{block}-p}\left(i\right)$ denotes the average pixel value of a neighborhood block and ${\bar{g}}_{\text{block}-c}=1/s^{2}\sum_{i=1}^{s^{2}}g_{\text{block}-c}\left(i\right)$ represents the average pixel value of a center block. In equation (1), the function *t*(·) is defined as

(2) $$\begin{array}{c} t\left({\bar{g}}_{\text{block}-p}-{\bar{g}}_{\text{block}-c}\right)=\left\{\begin{array}{cc} 1, & {\bar{g}}_{\text{block}-p}\geq {\bar{g}}_{\text{block}-c,}\\ 0, & {\bar{g}}_{\text{block}-p}<{\bar{g}}_{\text{block}-c.} \end{array}\right. \end{array}$$

**Figure 3:**
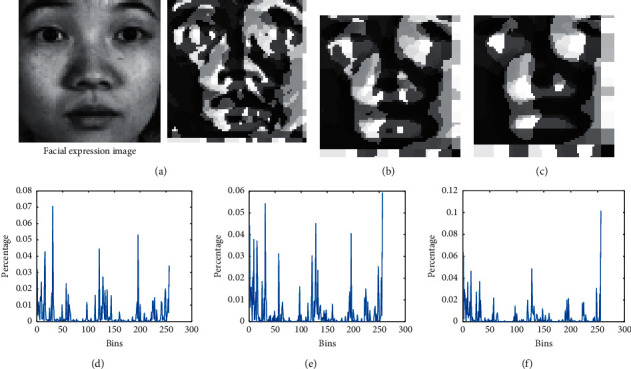
MB-LBP feature images and their corresponding uniform histograms. (a), (b), and (c) MB-LBP feature images with scale parameter *s* = 3, 5, and 7, respectively. (d), (e), and (f) Uniform histograms corresponding to (a), (b), and (c), respectively.

In Figure 3, the influence of the scale parameter *s* is great, and the macrostructure and microstructure of the expression texture are exhibited well. In Figures 3(a)–3(c), as the size of parameter *s* increases, the noise in the regions filtered by MB-LBP decreases, which represents the expression structure more robustly. Therefore, if parameter *s* is selected properly, then it contributes to reducing the extrapersonal differences, while at the same time, it highlights the representation of expression features.

However, the MB-LBP image has the form of a two-dimensional matrix. When transforming a 2D MB-LBP matrix into a one-dimension vector, high-dimensional data will be produced. To address the dimension of the MB-LBP matrix and retain the all the information of the structure, we normalize the MB-LBP matrix to a uniform histogram pattern, which fixes the dimension into a relatively low-dimension pattern while not losing any information.

The uniform histogram pattern of the MB-LBP is defined by the following steps. First, according to the grayscale level of the pixels of an image, an MB-LBP feature image is partitioned into 256 bins from 0 to 255. Second, the number of MB-LBP image pixels is counted according to grayscale level in increasing order. Then, these statistical numbers of the MB-LBP pixels are put into bins from 0 to 255. Finally, when given an *m* × *n* MB-LBP feature image, the MB-LBP uniform histogram is described as follows:

(3) $$\begin{array}{c} \left\{\begin{array}{c} f\left(x,y\right)=p_{i},\;i=0,1,2,\ldots ,255,\\ \text{his}\left[\cdot \right]=\sum f\left(x,y\right),\;x\in R^{m},\;y\in R^{n}, \end{array}\right. \end{array}$$

where *f*(*x*, *y*) is the pixel value corresponding to each grayscale level and his[*·*] is the uniform histogram feature number. The MB-LBPUH is normalized as follows:

(4) $$\begin{array}{c} {\text{MB}}_{\text{hist}}=\frac{\text{his}\left[\cdot \right]}{m\times n}. \end{array}$$

#### 3.2.2. HOG Feature Extraction

**Figure 4:**
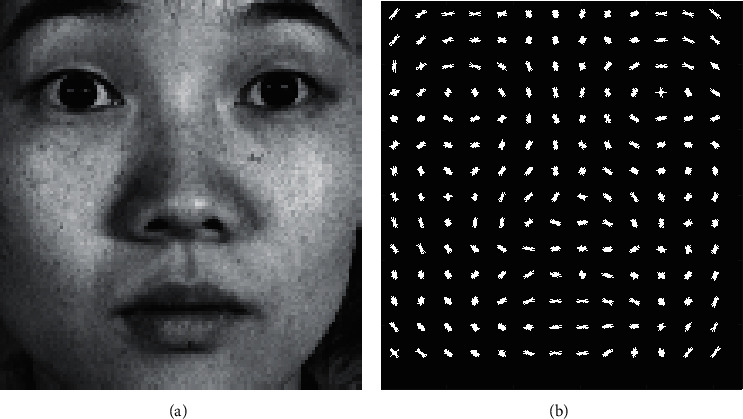
HOG feature image. (a) Facial expression image. (b) HOG feature visualisation.

The HOG representation was first proposed for human detection [18]. This representation is based on the statistical distribution of the local intensity gradients or edge directions that characterize the appearance and shape of a local object well. The HOG descriptor has some accumulation operations. The main operation is accumulating a local histogram of gradient directions or edge orientations after dividing an image into a set of small spatial regions named cells. The other operation is accumulating a measure of the local histogram over somewhat larger spatial regions named blocks. A block is composed of cells, and the accumulated results are used to normalize all the cells in the block.  Figure 4 shows an HOG facial expression image that exhibits a local texture appearance and shape.

**Figure 5:**
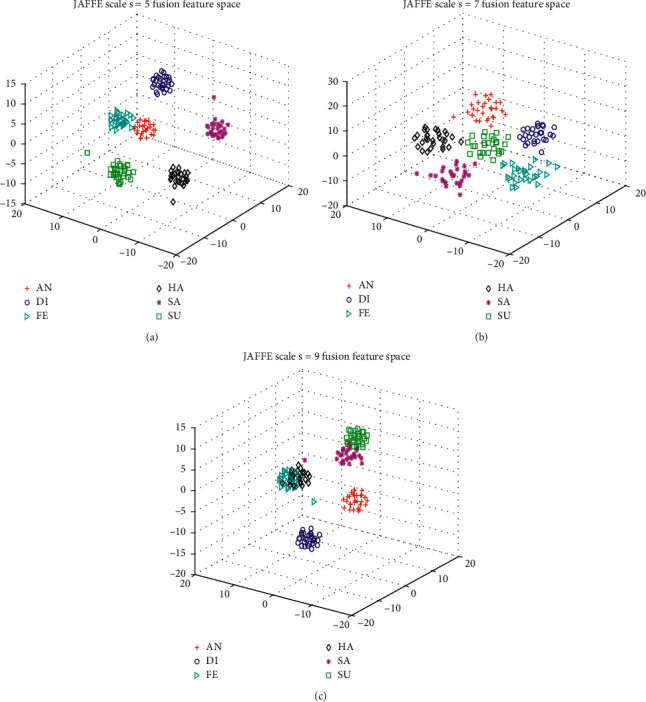
3D feature plots of the fusion features in multiparameter LDA spaces on the JAFFE dataset. (a), (b), and (c) Fusion features in the LDA feature space with scale *s* = 5, *s* = 7, and *s* = 9, respectively.

As previously mentioned in Section 3.2.1, facial expression peculiarities can be characterized well once the parameters of MB-LBPUH are appropriately selected. The selection of the MB-LBPUH scale parameter is based on the cross-validation recognition accuracy of MB-LBPUH filtering over training samples. In the context of the selected parameters, we build multiparameter LDA fusion feature spaces in which an MB-LBPUH feature and an HOG feature are concatenated to form a fusion feature.  Figure 5 constructs three LDA fusion feature spaces with the selected parameter *s* = 5, 7, and 9 according to the three best cross-validation recognition accuracies of the JAFFE dataset.

### 3.3. Decision Voting Strategy for Prediction

Majority voting is a simple and effective decision-level data fusion method. Majority voting utilizes multiple classifiers to identify the category of a test sample and then selects the most votes of a particular class as a prediction output. However, employing multiple classifiers for decision fusion increases the complexity of the model, especially when conducting classification in multiple spaces. In our model, we propose a simple decision voting strategy in multiple spaces to predict the categories of facial expressions. As previously mentioned, decision voting is applied to build spaces according to the MB-LBPUH parameters. If the votes of a particular class are the most overall spaces, then the class is identified as a predicted result. The Nearest Neighbors (NN) classifier is a particular case of the *k*-Nearest Neighbors (KNN) classifier. The essential principle of the NN classifier is calculating the distances between a new sample and known class samples and predicting the label based on the nearest distance.

To maintain the robustness of the NN classification and eliminate the influence of abnormal data, we use the distance between a new sample and the center point of each class as the measure. In other words, when a test sample is input, if it has the nearest distance to the center of a certain class, then it will belong to the class. Therefore, the NN-based decision voting rule of our model is the following: a test sample is first categorized by the NN classifier in multiparameter LDA feature spaces, and then voting is conducted in each space. If more than half of the votes predict that the sample belongs to the same class, then the sample is identified as the correct one.

### 3.4. Computational Complexity

The proposed feature fusion model mainly includes three algorithms, i.e., the MB-LBPUH and HOG feature extraction algorithms and the LDA dimension reduction algorithm. Subsequently, analysis of the computational costs of these algorithms is performed as follows.

The main idea of the MB-LBPUH algorithm is to compare the average grayscale values of neighboring blocks to the average grayscale values of the center block. Therefore, when given an *m* × *n* image, the computational complexity is *O*(*mn*)+*O*(1). In HOG feature extraction, the major computation is calculating the gradient intensity and direction over the pixels of a whole image region with the computational cost *O*(*mn*). If the size of the HOG cell and block is defined as *c* × *c* and *b* × *b*, respectively, then the computational cost of calculating the histogram of cells is *O*(*c*^2^) and that of the histograms of cells in blocks is *O*(*b*^2^). The total computational cost of the HOG algorithm is *O*(*mn*)+*O*(*c*)^2^+*O*(*b*)^2^ for *b* < *c* < min(*m*, *n*) in practice. In addition, the calculation cost of LDA is *O*(*C*) with *C* classes.

## 4. Experiments and Discussion

### 4.1. Datasets and Image Reprocessing

**Figure 6:**
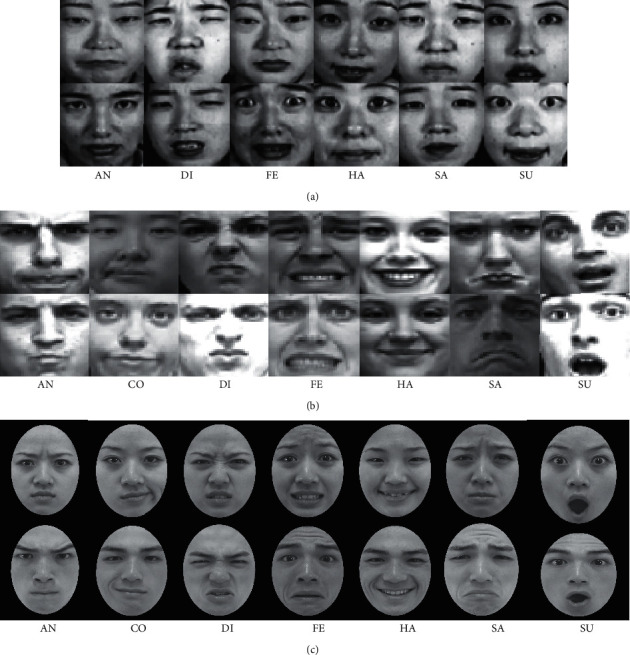
Facial expression images of JAFFE, CK+, and TFEID datasets. (a) JAFFE. (b) CK+. (c) TFEID.

In the experiments, we use three mainstream databases to evaluate the performance of the proposed model. The Japanese Female Facial Expression (JAFFE) database contains 10 female subjects including 6 basic facial expressions: anger (AN), disgust (DI), fear (FE), happiness (HA), sadness (SA), and surprise (SU). There are three or four images in each class, and the total number of sample images is 183. The extended Cohn-Kanade (CK+) database [60] includes 539 image sequences from 123 subjects. These sequences describe the changes of facial expressions from neutral to peak, and the last frame is commonly taken as an expression image used as a sample image. In the CK+ database, seven basic facial expressions are utilized for training and testing, including anger, contempt (CO), disgust, fear, happiness, sadness, and surprise, with a total of 327 images. The TFEID dataset [61] is composed of 7200 stimuli captured from 40 models aged between 18 and 30 years. This dataset contains seven types of facial expressions except for neutral expressions. We list the number of images and facial expression classes in Table 1.  Figure 6 shows some sample images of the three datasets.

It is important to mention that there are 327 samples in the CK+ dataset. The number of samples in each class is imbalanced, which leads to frustrating results [62]. Thus, we resampled the images into smaller classes and downsampled images into larger classes. Further, in smaller classes, three or four frames of a sequence (not only peak frames) were used as samples. Before feature extraction, we cropped the face images according to the eye location and resized them to 64 × 64 without any other image preprocessing.

**Figure 7:**
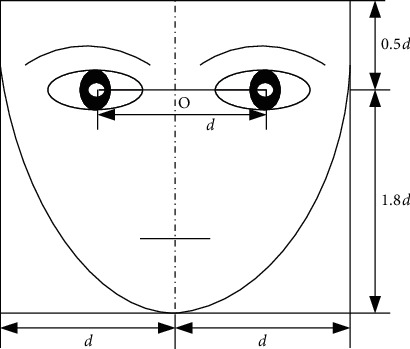
Face geometric normalization model.

In image preprocessing, image cropping is a geometric normalization method employed to normalize the size of images. The method is as follows:

  Step 1. Manually determine the coordinates of the center points of both eyes.
  Step 2. According to the distance of the center points of both eyes, crop the face image in the horizontal and vertical directions. The size of the cropping of an image is illustrated in detail in Figure 7, which is adjusted to maximize the retention of the facial expressions of face images.

### 4.2. MB-LBPUH Parameter Selection

A bright spot of the paper is the parameter selection of MB-LBPUH, which well characterizes the structural changes of facial expressions. The standard for assessing MB-LBPUH parameter selection is the cross-validation recognition accuracy. Further, we set the parameter *s* at 3 × 3,5 × 5,…, (2*n*+1) × (2*n*+1) and then used MB-LBPUH with these parameters to filter the expression images. The whole parameter selection experiment was implemented by using nested cross-validation with seven MB-LBPUH parameters (3, 5, 7, 9, 11, 13, and 15) in the training samples. In the nested cross-validation, the outer is 5-fold cross-validation and the inner is 10-fold cross-validation. The average result of the cross-validation is used as the final result.

**Figure 8:**
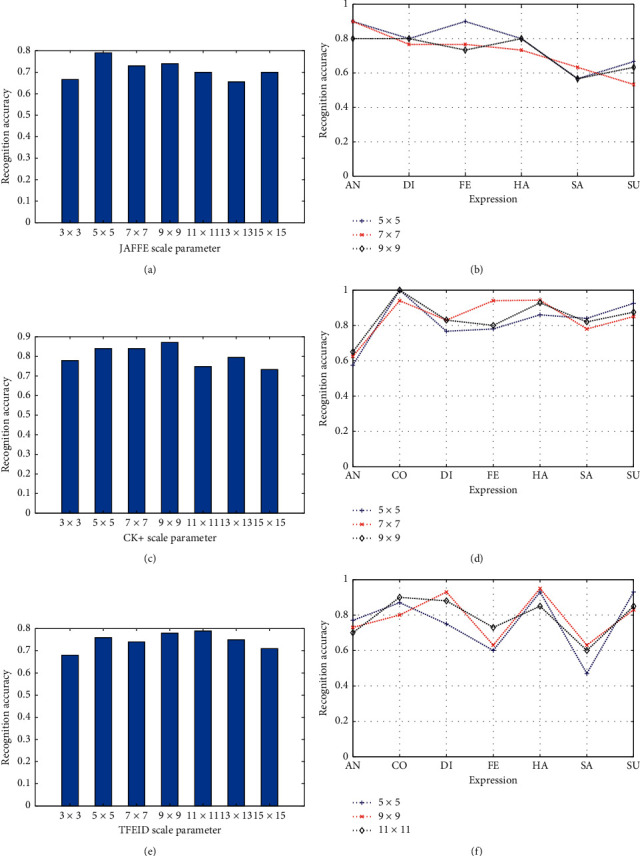
Selection of the MB-LBPUH parameters on the JAFFE, CK+, and TFEID datasets. (a), (c), and (e) The cross-validation accuracies of MB-LBPUH. (b), (d), and (f) The recognition accuracies of the various expressions with the selected parameters on the JAFFE, CK+, and TFEID datasets, respectively.

Figure 8 illustrates the selection results of the MB-LBPUH parameter on three datasets. We experimentally observe that the optimal parameters of different expressions are distributed in these scales, which have the three best cross-validation accuracies of MB-LBPUH. That is, in the CK+ dataset, as observed in Figure 8(c), the excellent performances of the MB-LBPUH features of different expressions are distributed in the three best cross-validation accuracies with scales *s*=5 × 5,7 × 7,  and 9 × 9. However, to further observe the results of the experiments (Figure 8(d)), the performances of the CO, FE, and SU expression features extracted by MB-LBPUH corresponding to *s*=9 × 9,7 × 7,  and 5 × 5, respectively, are superior to the cases of others.

A similar case also was also found in the JAFFE and TFEID datasets. That is, the excellent structural performance of the expressions can be characterized discriminatively by the MB-LBPUH operator with the selected parameters. Therefore, according to the experimental demonstration, the optimal parameters of the three datasets were obtained with *s*=5 × 5,7 × 7,  and 9 × 9 on the JAFFE dataset; *s*=5 × 5,7 × 7,  and 9 × 9 on the CK+ dataset; and *s*=5 × 5,9 × 9,  and 11 × 11 on the TFEID, respectively. Accordingly, to better reflect the feature representation, we then built multiparameter feature spaces for classification.

### 4.3. Experimental Setting and Discussion

The experiments were designed based on the MATLAB R2017b environment. Comparative experiments were conducted by employing the 10-fold cross-validation strategy to evaluate the performances of various feature models.

In the first part of the comparative experiments, we employed an SVM classifier with a linear kernel to predict the testing samples. Several traditional feature extraction algorithms were also performed with 10-fold cross-validation, namely, the Gabor wavelet, LBP, and HOG algorithms. The uniform pattern of LBP was utilized for feature extraction, and the dimension was 59. Gabor was used to extract features from five scales and eight directions, which resulted in a high dimension of 163,840. In addition, the dimension of the extracted HOG was 1,764. To address these high-dimension data, we employed PCA to reduce the dimensions.

Tables 2–4 list the comparison results between the traditional feature extraction methods and the proposed method on the JAFFE, CK+, and TFEID datasets. As a single feature extraction method, HOG performed better than other traditional methods. It is through the statistical computation of the distribution of local intensity gradients or edge directions that HOG characterizes the local object appearance and shape rather well. However, it is worth noting that the single feature extraction method is not applied effectively on each class. For example, as shown in Tables 2–4, the expression recognition (ER) accuracies of the Gabor features in the FE and SA classes are far less than those in other expression classes. Notably, in Table 3, the highest recognition accuracy of the Gabor feature of the CO class is 95.64%, and the lowest one in the SA class is 57.42%. The difference between the highest and lowest recognition accuracies is more than 30%.

Other traditional methods have the same case: the ER accuracy of LBP is 77.67% on the SU class, but it drops to 54.17% and 57.50% on the FE and SA classes, respectively. Although the HOG descriptor exhibits good performance, it is also invalid in some expression classes. That is, as shown in Table 3, the ER accuracy of HA reaches up to 100%, but the ER accuracy of AN drops by 17.78%. Similar cases are listed in Table 4. The differences of the ER accuracies among different classes are obvious; even the difference between the highest ER accuracy and the lowest ER accuracy is more than 35%.

Therefore, a single traditional method cannot accurately recognize all the expression classes. One of the reasons is that some expressions are difficult to distinguish, such as AN, FE, and sadness. Furthermore, finding the right feature extraction approach is extremely important for facial expression recognition. In the proposed model, MB-LBPUH with appropriate parameters excellently describes the structural peculiarity of different emotion expressions. The fusion feature of MB-LBPUH combined with HOG leads the ER accuracy of each expression to be more stable, and the maximum difference between two similar classes, such as the AN and FE classes, listed in Table 4 is no more than 11%. The ER accuracies of the proposed method in Tables 2–4 are the best: 94.58%, 98.21%, and 93.50%, respectively. In addition, Tables 5–9 list the comparison results of the various methods including hand-crafted and deep learning applied to the JAFFE, CK+, and TFEID datasets, respectively. Especially, as shown in Tables 6 and 8, the proposed model outperforms some state-of-the-art deep learning approaches. The essential reason for the better performance of the proposed model is that the proposed fusion feature characterizes the structural and textural features of expressions in detail. Combining them can represent facial expression excellently. The quantitative comparison also indicates that our model has superior results compared with the other models.

In the second comparative experiment, to further demonstrate the performance of the proposed model in different spaces, we constructed multiparameter PCA feature spaces in which the feature fusion and decision voting strategy were similar to our proposed model. To illustrate the advantage of the proposed method, we compared NN-based decision voting with various classifiers (KNN, SVM, and Sparse Representation-based Classification (SRC) [76]) for prediction. To conduct a fair comparison, the parameters of the classifiers were set as follows: *k* = 3 for the KNN, and the kernel function of the SVM was the linear kernel function.

**Figure 9:**
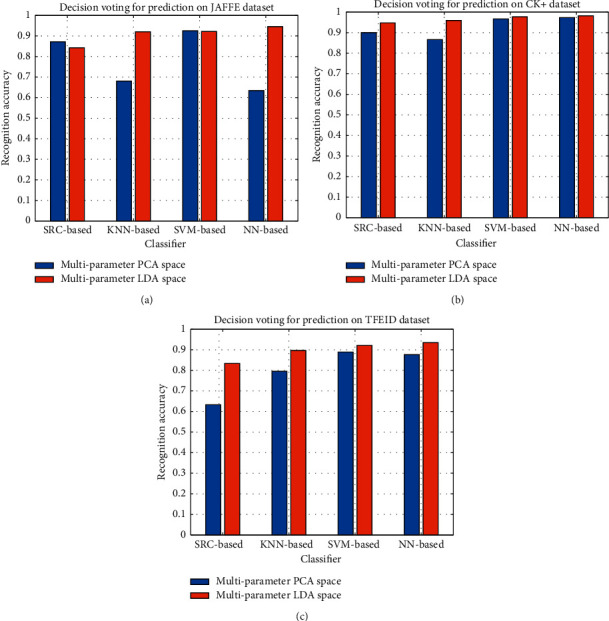
Prediction result comparison of the multiparameter PCA and LDA feature spaces on the (a) JAFFE, (b) CK+, and (c) TFEID datasets, respectively.

Figure 9 illustrates the performance comparison of various decision voting-based classification strategies in multiparameter PCA feature spaces and multiparameter LDA feature spaces. In most cases, the prediction results in the LDA space are better than those in the PCA space. Except for the NN-based decision, the prediction performance of the SVM-based decision is better than that of other KNN-based and SRC-based decision strategies. This is because the SVM uses a nonlinear kernel function to handle the data attribution. However, the SVM algorithm is more complicated than the NN algorithm. Furthermore, the LDA pays attention to the differences of known categories, which provides convenience for pattern classification. Hence, all kinds of samples are projected into the LDA space by maximizing the interclass differences and minimizing the intraclass differences. In context of LDA, the distance measure of the NN classifier between an unknown sample and the center point of each class is stable and effective. Even more critically, it has low computational complexity.

### 4.4. Further Discussion

In our current research, the proposed multiple feature fusion model can both enhance feature discrimination and resolve the inconvenient classifiable pattern problem. The proposed multiple feature fusion model achieves superior performance compared to some state-of-the-art approaches. However, the proposed method has some possible limitations that need to be addressed.

First, in the LDA space, the fusion feature is composed of MB-LBPUH and HOG features, which contain a considerable amount of redundant information. To obtain a discriminative feature, a feature selection method needs to be explored to obtain an excellent feature representation. Second, although the fusion feature reflects the structural information and local textural information, it lacks consideration of the evaluation measure of the two selected features. In future work, we will design an evaluation measure of how to select two or more features for feature fusion. Finally, multiple feature fusion should be considered in a video sequence, which will make the research work more practical. For example, the emotion of a speaker can be recognized through a fusion feature composed of gesture features, dynamic texture features, and dynamic geometric deformation features.

## 5. Conclusions

In the past decade, most research work on FER aimed at achieving perfect ER accuracy. Many improved pattern recognition models have been developed, i.e., a single feature extraction that combines various classification strategies and two or more kinds of features fused together to characterize the essential object features better. In our work, before feature extraction, the images were only preprocessed by cropping and resizing, without conducting any other image preprocessing. The highlight of the paper is the MB-LBPUH parameter selection. Once the appropriate parameters are defined, the extracted MB-LBPUH features vividly characterize the structural changes of expressions. MB-LBPUH parameter selection is based on the cross-validation accuracy of MB-LBPUH. Experiments demonstrate that various facial expressions have the best representation using the selected parameters.

Based on the textural particularity of expressions, the gradients and directions are described preferably to characterize expression features. Accordingly, the HOG descriptor performs this work well. MB-LBPUH and HOG features are fused together for feature extraction. These features not only contain holistic structural information but also contain local textural information. It is worth noting that we built the multiparameter LDA feature space. An unknown sample could be projected into LDA spaces to find its best match, and then, decision voting could be used to predict the category of the sample. In general, the proposed FER model exhibited superior performance compared to existing approaches on the JAFFE, CK+, and TFEID datasets; the ER recognition accuracies were as high as 94.58%, 98.21%, and 93.50%, respectively. As a future research direction, we will focus on feature selection, the feature fusion model, and deep neural networks in facial expression recognition.

## Figures and Tables

**Table 1:** Number of images and facial expression classes of different datasets.

Dataset	AN	CO	DI	FE	HA	SA	SU
JAFFE	30	—	29	32	31	31	30
CK+	77	71	73	74	69	76	75
TFEID	33	40	40	40	40	39	36

**Table 2:** Comparison of the ER accuracy (%) of various features on the JAFFE dataset.

Method	AN	DI	FE	HA	SA	SU	ER accuracy (%)
LBP + SVM	60.00	73.33	54.17	63.33	57.50	77.67	64.33
HOG + SVM	93.33	87.67	87.86	94.52	86.67	93.33	90.56
Gabor + SVM	93.33	80.67	77.33	91.17	66.67	86.00	82.53
The proposed method	93.33	91.67	95.00	100.00	94.17	93.33	94.58

**Table 3:** Comparison of the ER accuracy (%) of various features on the CK + dataset.

Method	AN	CO	DI	FE	HA	SA	SU	ER accuracy (%)
LBP + SVM	83.22	100.00	95.12	93.80	100.00	94.18	92.03	94.05
HOG + SVM	82.22	95.00	91.36	92.00	100.00	100.00	90.00	92.94
Gabor + SVM	83.38	95.64	83.03	60.00	89.78	57.42	95.22	85.76
The proposed method	93.39	100.00	94.29	100.00	95.71	98.75	98.75	98.21

**Table 4:** Comparison of the ER accuracy (%) of various features on the TFEID dataset.

Method	AN	CO	DI	FE	HA	SA	SU	ER accuracy (%)
LBP + SVM	60.00	96.17	99.41	71.97	98.42	68.90	100.00	84.98
HOG + SVM	88.97	96.67	96.14	82.43	97.89	87.17	97.42	92.38
Gabor + SVM	82.38	94.64	82.97	83.08	93.14	64.12	100.00	85.76
The proposed method	90.33	100.00	93.50	89.99	95.00	90.67	95.00	93.50

**Table 5:** Comparisons with various feature extraction approaches on the JAFFE dataset.

Ref.	Feature	Evaluation	Classification	ER accuracy (%)
2010 [63]	Normalized image	Leave-one-out	GP classifier	93.00
2012 [64]	DKLLE	10-fold	SVM	84.17
2016 [65]	2DPCA	Person-dependent	RF	93.83
2017 [66]	Pyramid + CS	10-fold	SBDT SVM	91.43
2017 [67]	LBP + HOG	10-fold	SVM	90.00
The proposed method	Fusion feature	10-fold	Decision voting	94.58

**Table 6:** Comparisons with the state-of-the-art deep learning approaches on the JAFFE dataset.

Ref.	Method	Average (%)
2015 [68]	Sobel-CNN	92.60
2017 [69]	CNN	84.48
2018 [54]	WMDNN	92.21
2019 [57]	Hierarchical network	91.27
The proposed method	Fusion feature	94.58

**Table 7:** Comparisons with various feature extraction approaches on the CK+ dataset.

Ref.	Feature	Class	Evaluation	Classification	ER accuracy (%)
2012 [70]	Common + specific patch	6	10-fold	SVM	88.25
2013 [71]	LBP + geometric feature	6	5-fold	SVM	89.56
2014 [45]	LPQ + PHOG	7	7-fold	SVM	93.21
2017 [72]	Data-driven	7	Leave-one-out	SVM	94.81
The proposed method	Fusion feature	7	10-fold	Decision voting	98.21

**Table 8:** Comparisons with the state-of-the-art deep learning methods on the CK+ dataset.

Ref.	Method	Average (%)
2015 [59]	DTAGN (joint)	97.25
2016 [73]	RBM	95.66
2018 [54]	WMDNN	97.02
2019 [55]	DCMA-CNNS	93.46
2019 [57]	Hierarchical network	96.46
The proposed method	Fusion feature	98.21

**Table 9:** Comparisons with various approaches on the TFEID dataset.

Ref.	Feature	Class	Evaluation	Classification	ER accuracy (%)
2014 [74]	MPC-based	7	10-fold	SVM	92.54
2017 [75]	Haar wavelet	7	10-fold	LR	89.58
2017 [66]	Pyramid + CS	7	10-fold	SBDT SVM	93.38
The proposed method	Fusion feature	7	10-fold	Decision voting	93.50

## Data Availability

The data used to support the findings of this study are included in the article.
